# North Ormesby Hospital Enlarged

**Published:** 1924-11

**Authors:** 


### NORTH ORMESBY HOSPITAL ENLARGED.

As a result of the generous response to an appeal for money for the extension of the North Ormesby Hospital, Middlesbrough, by which £22,000 was collected, an additional wing was opened on October 20 by Sir Hugh Bell, Bt., according to the design of Mr. James Forbes. The new wing holds forty cots for children on the ground floor and thirty beds for women above, and is from a hygienic point of view a good deal ahead of the older parts of the building. From the beginning of the Hospital, some sixty years ago, it has been in the hands of the Sisters of the Community of the Holy Rood, whose capacity has made it to-day one of the two big general hospitals of Middlesbrough and a recognised training school for nurses, with, including the new wards, 176 beds. The Sisters are helped in the management by a governing body of twenty-seven who meet monthly. The hospital removed to its present site in one of the pleasantest parts of the town in 1861, where, under the chairmanship of Mr. J. H. Amos and the secretaryship of Mr. H. Cuthbert Hopkins, it continues to grow rapidly.

### Butter Instead of Margarine.

Dr. P. V. Davies has reported to the Kingston Guardians the large increase in the number of patients (including thirty-nine private patients) during the past half-year, which has involved the use of the nurses' recreation room. A more satisfactory fact is the decision of the Guardians to give the patients butter instead of margarine. Though the male observation wards are still in charge of the master, the Ministry of Health's suggestion that they be transferred to the control of the medical officer has not been opposed by the House Committee which has called upon the master to report.

